# Transcranial Electromagnetic Treatment “Rebalances” Blood and Brain Cytokine Levels in Alzheimer’s Patients: A New Mechanism for Reversal of Their Cognitive Impairment

**DOI:** 10.3389/fnagi.2022.829049

**Published:** 2022-05-02

**Authors:** Chuanhai Cao, Haitham Abulaban, Rob Baranowski, Yanhong Wang, Yun Bai, Xiaoyang Lin, Ning Shen, Xiaolin Zhang, Gary W. Arendash

**Affiliations:** ^1^Taneja College of Pharmacy, University of South Florida, Tampa, FL, United States; ^2^MegaNano Biotech, Inc., Tampa, FL, United States; ^3^Axiom Clinical Research, Tampa, FL, United States; ^4^University of South Florida Health Byrd Alzheimer’s Institute, Tampa, FL, United States; ^5^Left Coast Engineering, Escondido, CA, United States; ^6^NeuroEM Therapeutics, Inc., Phoenix, AZ, United States

**Keywords:** Transcranial Electromagnetic Treatment, Alzheimer’s disease, cytokines, immunoregulation, brain and blood

## Abstract

**Background:**

The immune system plays a critical role in the development and progression of Alzheimer’s disease (AD). However, there is disagreement as to whether development/progression of AD involves an over-activation or an under-activation of the immune system. In either scenario, the immune system’s cytokine levels are abnormal in AD and in need of rebalancing. We have recently published a pilot clinical trial (https://clinicaltrials.gov/ct2/show/NCT02958930) showing that 2 months of daily in-home Transcranial Electromagnetic Treatment (TEMT) was completely safe and resulted in reversal of AD cognitive impairment.

**Methods:**

For the eight mild/moderate AD subjects in this published work, the present study sought to determine if their TEMT administration had immunologic effects on blood or CSF levels of 12 cytokines. Subjects were given daily in-home TEMT for 2 months by their caregivers, utilizing first-in-class MemorEM™ devices.

**Results:**

For eight plasma cytokines, AD subjects with lower baseline cytokine levels always showed increases in those cytokines after both a single treatment or after 2-months of daily TEMT. By contrast, those AD subjects with higher baseline cytokine levels in plasma showed treatment-induced decreases in plasma cytokines at both time points. Thus, a gravitation to reported normal plasma cytokine levels (i.e., a “rebalancing”) occurred with both acute and long-term TEMT. In the CSF, TEMT-induced a similar rebalancing for seven measurable cytokines, the direction and extent of changes in individual subjects also being linked to their baseline CSF levels.

**Conclusion:**

Our results strongly suggest that daily TEMT to AD subjects for 2-months can “rebalance” levels for 11 of 12 cytokines in blood and/or brain, which is associated with reversal of their cognitive impairment. TEMT is likely to be providing these immunoregulatory effects by affecting cytokine secretion from: (1) blood cells traveling through the head’s vasculature, and (2) the brain’s microglia/astrocytes, choroid plexus, or neurons. This rebalancing of so many cytokines, and in both brain and systemic compartments, appears to be a remarkable new mechanism of TEMT action that may contribute substantially to it’s potential to prevent, stop, or reverse AD and other diseases of aging.

## Introduction

During aging, the modulation of immune responsiveness becomes less tightly regulated (Rea et al., 2019). This dysregulation of cytokine “balance” between pro-inflammatory and anti-inflammatory immune components is a common characteristic of aging and age-related diseases (Chung et al., 2009; Franceschi and Campisi, 2014). The role played by the immune system in Alzheimer’s disease (AD) pathogenesis is not limited to the brain’s microglia and astrocytes (Newcombe et al., 2018; Thawkar and Kaur, 2019), but also includes immune function within the systemic vasculature (Leung et al., 2013). The latter contains white blood cells (WBCs) that secrete a variety of cytokines/growth factors, as do the brain’s microglia and astrocytes – these cytokines/growth factors are the innate immune system’s effectors and modulators.

Alzheimer’s disease is often described as a disease of brain and/or peripheral inflammation, involving an “over-activation” of the immune system and ensuing elevated cytokine levels in brain (Hu et al., 2019) and/or blood (Fila, 2010; Rubio-Perez and Morillas-Ruiz, 2012; Sopova et al., 2014; Webers et al., 2020). Thus, many AD researchers believe that AD, at least in part, is caused by a low-grade inflammation in brain and/or systemically/peripherally (Candore et al., 2010) and that administration of agents that lower high cytokine levels in the brain parenchyma or in the vasculature could be therapeutic. However, general anti-inflammatory agents, such as NSAIDs and prednisone, have failed to show clinical benefits in AD patients (ADAPT-FS Research Group, 2015), as have agents purported to reduce brain microglial activity (ClinicalTrials.gov Identifier: NCT02080364 and NCT02916056).

There is also evidence for AD as a disease involving “insufficient activation” of the immune system, resulting in an inability to resist AD pathogenesis and the progressive cognitive impairment it provides – especially in the years prior to AD diagnosis when mild cognitive impairment (MCI) is present. For example, we have found that plasma levels of three cytokines (GCSF, IL-6, and IL-10) are reduced in MCI patients that convert to AD, but not in those that remain in MCI (Cao et al., 2012). These findings suggest that immune decline occurs in the years prior to AD diagnosis. In AD patients, low levels of GCSF and IL-15 have been reported (Renzos et al., 2007; Laske et al., 2009) and faster disease progression was found in AD patients with low plasma cytokine levels compared to those with high levels (Taipa et al., 2019). Conversely, individuals with Rheumatoid Arthritis have high levels of multiple cytokines in their plasma (Burska et al., 2014) and an associated reduced risk of developing AD (McGeer et al., 1996). Therapeutically, Potter and colleagues have reported improved mini-mental state exam (MMSE) scores in AD patients following 3-weeks of GMCSF injections (Potter et al., 2021). Thus, AD may involve a “hypoactive” immune system at some stage(s) in its pathogenesis, with the subjects’ low blood cytokine levels making them more susceptible to AD development and progression. Under this scenario, AD subjects with a hypoactive immune system could benefit from a therapeutic that activates (increases) cytokine levels in the blood or bolsters immune function within the brain.

In either over-activated or under-activated scenarios, the immune system’s cytokine levels are imbalanced in blood/brain and thus in need of “rebalancing” (Taipa et al., 2019). As such, a therapeutic that could provide “overall” regulation to blood and brain/CSF cytokine levels in AD subjects, such that cytokines return to the normal or near normal levels of aged unimpaired individuals, could contribute to any improvement in disease symptoms observed and possibly arrest or reverse disease progression. Unfortunately, no such “overall” immunoregulatory or “rebalancing” therapeutic is available, with AD researchers primarily developing drugs that target a single cytokine, such as TNF-α or targeting microglial modulation (Thawkar and Kaur, 2019).

Transcranial Electromagnetic Treatment (TEMT) is a new bioengineering-based neuromodulatory approach that we have been clinically developing against AD that involves brain treatment with electromagnetic (radiofrequency) waves – not to be equated with magnetic-based neuromodulatory approaches such as Transcranial Electromagnetic Stimulation (tMS) or Pulsed Electromagnetic Stimulation (PEMF). Our comprehensive pre-clinical studies in AD transgenic mice have shown that electromagnetic waves/fields “disaggregate” small intra-neuronal aggregates/oligomers of two toxic proteins that appear to be at the root causes of AD – Aβ and tau (Arendash et al., 2010, 2012; Dragicevic et al., 2011; Mori and Arendash, 2011; Arendash, 2012, 2016). These actions by TEMT, in combination with its ability to enhance brain mitochondrial function in neurons (Dragicevic et al., 2011), appear to be central to TEMT’s consistent ability to prevent or reverse cognitive impairment in AD transgenic mice (Arendash et al., 2010, 2012; Mori and Arendash, 2011).

To translate our pre-clinical findings to clinical trials in human AD subjects, we have developed a first-of-its kind head device, the MEMOREM™, for in-home TEMT administration to AD subjects by their caregiver. This unique device projects electromagnetic (radiofrequency) waves into the brain through multiple emitters distributed on the human head surface. These electromagnetic waves easily penetrate the human cranium and reach essentially all human forebrain areas (Arendash, 2016). We have recently published results from a comprehensive clinical trial that involved daily MEMOREM treatment to mild/moderate AD subjects (Arendash et al., 2019). Results from that trial indicated no deleterious side effects during a 2-month treatment period and reversal of cognitive impairment in key tasks (e.g., ADAS-cog and Rey AVLT). These published results also showed that TEMT can provide functional MRI (fMRI) benefits, and induce changes in Aβ isoforms within the CSF/plasma that are consistent with Aβ disaggregation in the brain (Arendash et al., 2019).

The present study investigated whether 2-months of daily TEMT administration to AD subjects in the aforementioned 2-month study (Arendash et al., 2019) had effects on their peripheral/vascular or brain immune systems, as indicated by changes in cytokine levels in plasma and CSF, respectively. To our knowledge, this is the first study investigating immune effects of TEMT or EMF treatment in humans, either acutely or long-term. Study results strongly suggest that TEMT can exert an overall immunoregulatory or “rebalancing” function in AD subjects for at least 11 prominent cytokines by regulating high or low cytokine levels – even following just a single treatment for plasma cytokines. Thus, immunoregulation or immune rebalancing is an important new mechanism of TEMT action against AD that can be added to its previously identified mechanisms of toxic oligomer disaggregation and mitochondrial enhancement.

## Materials and Methods

### Subjects

Eight subjects with mild-moderate AD were enrolled in this clinical trial over a period of late 2017 through mid-2018 at the University of South Florida Health Byrd Alzheimer’s Institute (Tampa, FL, United States). All subjects completed the 2-month treatment study by the end of 2018. Subjects had to be diagnosed with mild or moderate AD, according to the National Institute of Neurological and Communicative Disorders and Stroke-Alzheimer’s Disease and Related Disorders Association (NINCDS-ADRDA) criteria. All subjects were at least 63 years of age and exhibit an MMSE score of 16–26 at screening. In addition, subjects had to have a Global Deterioration Score above 2 and a Hachinski test score lower than 4. If subjects were being medicated with a cholinesterase inhibitor and/or memantine, they needed to be taking the medication(s) for at least 3 months prior to screening and maintained on their present dose throughout the period of this study. All subjects gave their consent to be in this study in accordance with informed consent regulations of the USF Health Byrd Alzheimer’s Institute.

Demographics and characteristics for each of the eight subjects participating in this study are shown in Table 1, with subjects being listed (left to right) according to their ADAS-cog score at baseline. Better performers (lower scores) are on the left and worse performers (higher scores) are on the right. In addition to the diagnosis of AD from the aforementioned cognitive assessment at screening/baseline, AD diagnosis was further substantiated by three indices included in this table, with detailed descriptions in our initial published results of this study (Arendash et al., 2019). First, anatomic MRIs at screening indicated the presence of frontal/parietal lobe atrophy, hippocampal/temporal lobe atrophy, and/or global cortical atrophy. Second, quantitative analysis of FDG-PET scans at baseline indicated glucose hypometabolism in three brain areas averaged to provide an “AD signature meta-ROI ratio” (Knopman et al., 2014), with an abnormal AD ratio being defined as ≤1.32 (90% AD sensitivity) (Jack et al., 2012, 2014). Third, the CSF Aβ1-42/t-tau ratio at baseline was calculated for each subject. The mean ratio of 0.90 is close to the mean ratio of 1.30 reported for diagnosed AD subjects, and far from the mean ratio of 3.96 for aged controls (Niemantsverdriet et al., 2017).

**TABLE 1:** Subject demographics/characteristics.

Subject	1	2	3	4	5	6	7	8	Mean
Age	77	76	66	63	65	74	82	63	70.8
Gender	F	F	M	F	F	F	M	F	–
ApoE Genotype	2/3	3/3	3/3	3/3	2/3	3/3	2/3	3/3	–
GDS Rating	4	4	3	5	4	3	4	4	3.9
Education (years)	19	16	15	13	14	16	12	14	14.9
Anat. MRI Analysis	b,c	b,c	b	c	b	a,b	b,c	a	–
PET AD Sign. ROI	1.10	1.06	1.10	1.32	1.40	1.25	1.06	1.32	1.20
Aβ1-42/t-tau Ratio	1.48	1.02	0.93	0.61	–	0.99	0.98	0.30	0.90
ADAS-cog13 Score	24	26.7	30.3	30.7	37.3	38.7	44.0	62.0	36.7

*a, frontal/parietal lobe atrophy; b, hippocampal/temporal lobe atrophy; c, global cortical atrophy.*

Both inclusion and exclusion criteria for this study are indicated in our initial published paper on this study (Arendash et al., 2019) and on the study’s clinicaltrials.gov website.^1^ For each subject, a caregiver (spouse, family member, etc.) having non-impaired mental abilities/motor skills needed to be identified to be responsible for administering daily treatments to the patient, keeping a diary of health measures they collect on the patient at home, and logging the patient’s condition daily.

### Investigational Device

**FIGURE 1:**
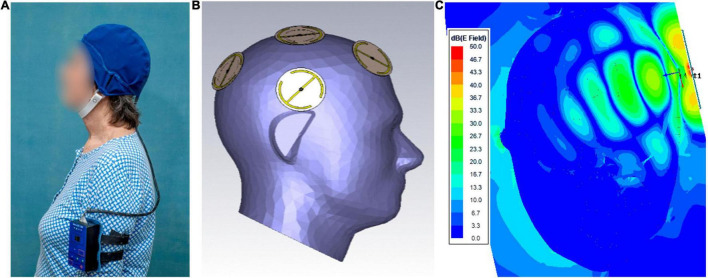
**(A)** A MemorEM™ device being worn by a subject. The control panel/battery box is worn on the upper arm and wired *via* a cable to eight electromagnetic emitters in the head cap. **(B)** Position of the eight electromagnetic emitters (four on each size of the head) embedded between the device’s two-layered head cap. Emitters collectively provide full forebrain TEMT *via* rapid sequential activation. **(C)** FDTD brain simulation of electric field showing penetration and distribution from a single emitter at a SAR power level of 1.6 W/kg. The head/emitter interface is the thin blue margin below the bow tie-like electric field generated by the emitter.

The MemorEM™ device has been designed for in-home daily treatment, allowing for complete mobility in performing most daily activities during treatment. The device has a custom-engineered circuit board and a rechargeable battery inside the box housing, as well as a control panel on the outside of the housing for treatment control. A custom-engineered circuit board/battery is contained within a box housing, which has a control panel on its surface. The box is worn on the upper arm and wired *via* a cable to eight emitters within a two-layered head cap (Figures 1A,B). Emitters are activated sequentially at 217 Hz such that only one emitter is active at any given time. When active, an emitter projects electromagnetic fields in the radiofrequency range into the brain at 915 MHz and 1.6 W/kg power level. At this frequency and power level, FDTD (ANSYS) human head computer simulations show that the eight emitters collectively provide penetrating TEMT to the human forebrain, including the cerebral cortex, underlying structures, and both superficial and deep cerebral vessels (Figure 1C). Though decreasing with penetration, computer simulated electric field levels are still around 20 V/m in the center of the human brain, as shown in Figure 1C. The MemorEM™ device and this clinical trial protocol were both approved as “non-significant risk” (NSR) by the Western Institutional Review Board (WIRB). In March 2020, the MemorEM device was designated by the FDA as its first “Breakthrough Device” for the treatment of AD.

### General Protocol

This clinical study was an open-label within-patient (single arm) single center study that was intended to evaluate the safety and efficacy of 2-months of daily TEMT administration in patients with mild-to-moderate AD. The initial publication of this study’s results (Arendash et al., 2019) involved endpoints of cognitive assessment, CSF and plasma AD biomarkers (e.g., Aβ and tau), brain FDG-PET quantitative analysis, and brain fMRI quantitative analysis. However, analysis of both CSF and plasma cytokine levels were not presented in that initial study due to its already comprehensive nature. Thus, the present study reports on the effects of 2-months of daily TEMT on both CSF and blood cytokine levels from that initial study. Reference to the original published study (Arendash et al., 2019) is made for screening and baseline events. For the purposes of the present study, 20 ml blood samples were collected at clinical visits for baseline, Day 1 (immediately following the first TEMT administration and 1 day following baseline), and after 2-months of twice-daily TEMT. CSF samples of 15 ml each were collected *via* spinal tap at clinical visits for baseline and after the 2-months of treatment. At baseline and following the 2-month treatment period, subjects were tested in a comprehensive battery of cognitive tasks, which included the principal measure of efficacy, the ADAS-Cog13 (maximum poor score of 85 points). The full protocol for this study is detailed at ClinicalTrials.gov (see text footnote 1) and provided in the initial report of findings (Arendash et al., 2019).

### Blood and CSF Cytokine Analysis

All 20 ml blood samples (collected at baseline and treatment Days 1 and 60) were divided into two 10 ml BD k_2_-EDTA tubes and centrifuged at 300 × *g* for 10 min. The plasma (upper layer) for each tube was transferred into a new 15 ml tube, then centrifuged at 2000 *g* for 10 min. One ml volumes of the top plasma layer were aliquoted into 1.5 ml tubes and stored at −80°C for future analysis. The two 15-ml samples of CSF collected at baseline and on Day 60 (2-months) were each aliquoted into 1.5 ml tubes, then frozen and stored at −80°C until analysis. Plasma/CSF samples were thawed completely on ice at the end of the study, with samples being mixed well on vortex and centrifuged at 2000 *g* for 10 min to precipitate any debris. Determination of plasma and CSF cytokine levels was performed in duplicate and averaged for the following 12 cytokines/growth factors: GCSF, GMCSF, VEGF, PDGF, IL-8, IL-10, IL-15, IL-17α, INFγ, and TGF-α were all detected by using multiplex kits from Millipore (Cat HCYTOMAg-60K), while NGF was detected by using a Millipore Adipokine magnetic bead panel kit (Cat HADK2MAG-61K) and IL-18 was detected by using a Singleplex magnetic bead panel kit from Millipore (Cat HIL18MAG-66K). All measurements were read on Bio-Rad Bio-Plex MAGPIX Reader.

### Statistical Analysis

This study’s time points of baseline (BL) and Day 60 (D60; end of treatment) were statistically analyzed for differences in cytokine levels between BL and D60, as well as between low BL and high BL sub-groups. Paired or unpaired *t*-tests were utilized to assess statistical significance of group or sub-group differences, with threshold for significance set at *p* < 0.05. In addition to *p*-value and given the limited number of subjects in the two “subgroups” of this study (*n*’s of 2–6 per sub-group), we also report more clinically relevant results comparing sub-groups in terms of Effect Size (ES) (Farivar et al., 2004), which is not dependent on number of subjects. ES measures the “magnitude of the difference between groups” and can be interpreted with regard to the minimal difference that is clinically important/meaningful, commonly set at a moderate effect reflecting Cohen’s *d* > 0.5. For determination of ES, the following established scale was utilized, based on Cohen’s *d* (Sawilowsky, 2009): moderate effect (>0.5), large effect (>0.8), very large effect (>1.2), huge effect (>2.0). For correlation analyses, correlation coefficients (*r*) were calculated and level of significant determined from Pearson’s correlation table. Data from a subject on a given measure was sometimes omitted due to undetectable baseline readings, inconsistent duplicate values, or as a clear outlier (Grubb’s single outlier test).

## Results

### Cognitive Results Summary

Since the cytokine levels in this study were attained from the same eight subjects in an already-published paper describing a reversal of cognitive impairment in AD subjects following 2-months of daily treatment (Arendash et al., 2019), a brief summary of those results is appropriate. Compared to baseline, overall ADAS-cog performance across 13 sub-measures and the ADAS-cog’s “Immediate Recall” component improved substantially. Similarly, AVLT 5-trial recall and “percent forgetting” were both improved, as was the number of digits remembered in the Digits Forward Length measure. This TEMT-induced reversal of cognitive impairment across multiple tasks/measures was maintained even 2 weeks following completion of treatment and was seen to the same degree in both male and female AD subjects alike.

### Alzheimer’s Disease Patients Divided Into Two Groups Based on Their Baseline Plasma Cytokine Levels

**FIGURE 2:**
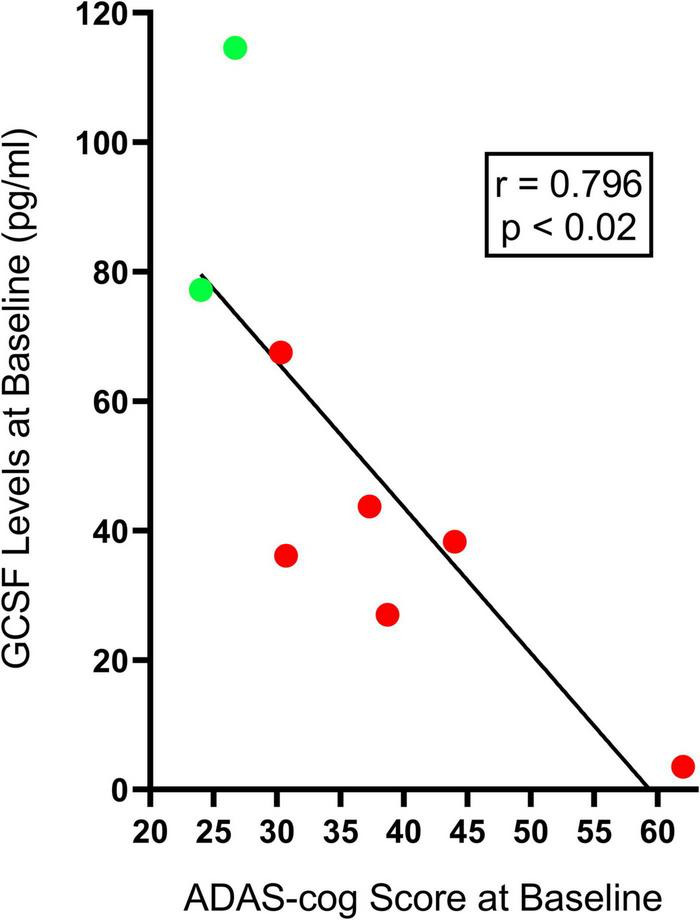
The significant inverse correlation between plasma baseline GCSF levels and baseline ADAS-cog scores in individual AD subjects. Lower baseline GCSF levels were evident in individual AD subjects having poorer (higher) baseline ADAS-cog scores (Group 1, red-filled circles), while higher baseline GCSF levels were found in individual AD subjects with better (lower) baseline ADAS-cog scores (Group 2, green-filled points). This inverse correlation was present for seven of the eight cytokines measures in plasma.

Table 2 shows the effects of 2-months of daily TEMT on overall levels of 12 cytokines in plasma, comparing baseline to levels on Day 60. For every cytokine measured in plasma, there was no “overall” change in levels induced by treatment. However, if baseline levels of each cytokine for all subjects were listed in rank order, they can be divided into lower and higher baseline cytokine groups – Groups 1 and 2, respectively (Table 3). For all of the 12 cytokines, comparison between lower vs. higher cytokine level groups *via t*-tests revealed 9 significant (*p* < 0.05) and 3 nearly significant differences. The same sub-group comparisons utilizing Effect Size indicated that all 12 comparisons of lower vs. higher baseline cytokine groups were different and of clinical important/meaningfulness (Table 3). It is noteworthy that the lower and higher cytokine level groups also differed from each other in baseline ADAS-cog score, with the lower baseline cytokine group having significantly poorer (higher) ADAS-cog scores compared to the higher baseline cytokine group for all cytokines except for IFN-γ. This is exemplified by the strong inverse correlation between baseline GCSF levels and baseline ADAS-cog scores in the same AD subjects (*r* = −0.796; *p* < 0.02) – lower baseline GCSF levels were present in subjects with poorer baseline ADAS-cog scores (Figure 2).

**TABLE 2:** Overall effects of 2-months TEMT administration on cytokines/growth factors in plasma (pg/ml).

Cytokine	Baseline	Day 60	*p*-Value	*n*
GCSF	50.98 ± 12.15	74.01 ± 10.71	0.193	8
GMCSF	17.01 ± 9.55	14.89 ± 4.92	0.714	8
VEGF	82.18 ± 36.36	152.96 ± 33.57	0.249	7
IL-10	12.61 ± 5.71	16.40 ± 5.22	0.518	7
IL-17α	2.59 ± 1.07	2.27 ± 0.40	0.741	6
IL-15	3.12 ± 2.00	4.79 ± 1.98	0.335	7
IL-18	2.77 ± 1.27	2.54 ± 0.31	0.856	6
PDGF	4,592 ± 1,777	7,941 ± 2,369	0.321	8
INFγ	4.02 ± 1.18	15.76 ± 11.12	0.324	7
TGF-α	0.60 ± 0.26	1.10 ± 0.47	0.457	7
IL-8	3.23 ± 1.43	5.42 ± 1.59	0.280	8
NGF	1.48 ± 0.15	1.67 ± 0.22	0.234	8

**TABLE 3:** Low vs. high baseline cytokine/growth factor levels in plasma (pg/ml).

Cytokine	Lower levels at baseline	Higher levels at baseline	*p*-Value	Cohen’s *d*	Effect Size
GCSF	36.0 ± 8.5 (6)	95.9 ± 18.1 (2)	**0.016**	2.76	Huge
GMCSF	1.71 ± 0.9 (5)	42.5 ± 18.1 (3)	**0.022**	2.24	Huge
VEGF	8.6 ± 6.2 (4)	180.3 ± 27.3 (3)	**0.0008**	4.97	Huge
IL-10	4.03 ± 1.69 (4)	24.04 ± 10.40 (3)	0.075	1.71	Very large
IL-15	0.29 ± 0.06 (4)	6.89 ± 3.95 (3)	0.102	1.53	Very large
IL-17α	0.27 ± 0.11 (3)	4.90 ± 0.62 (3)	**0.0018**	6.05	Huge
IL-18	0.28 ± 0.08 (3)	5.25 ± 1.37 (3)	**0.023**	2.95	Huge
PDGF	1725 ± 534 (4)	9371 ± 3203 (4)	**0.02**	1.72	Very large
INF-γ	2.71 ± 1.11 (5)	7.31 ± 1.29 (2)	0.068	1.94	Very large
TGF-α	0.11 ± 0.06 (4)	1.25 ± 0.30 (3)	**0.0074**	3.32	Huge
IL-8	2.13 ± 0.77 (6)	6.54 ± 5.83 (2)	**0.0081**	0.94	Large
NGF	1.20 ± 0.09 (5)	1.94 ± 0.15 (3)	**0.0036**	3.37	Huge

*Numbers in parenthesis indicate number of subjects. Individual values below assay sensitivity not included. For reference, “clinically important” effects are indicated by Effect Size using the following established scale of Cohen’s d (Sawilowsky, 2009): moderate effect (>0.5), large effect (>0.8), very large effect (>1.2), and huge effect (>2.0). Bold values represent significant comparison of lower verses higher.*

Given these two very different profiles of baseline cytokine levels and associated ADAS-cog performance, the effects of TEMT administration on plasma cytokine levels were appropriately evaluated in relation to baseline cytokine levels, with subjects divided into two groups for each cytokine – Group 1 had lower plasma cytokine levels (poorer cognitive performance) and Group 2 had higher plasma cytokine levels (better cognitive performance).

### Baseline Plasma Cytokine Levels Determine Direction of Response to 2 Months of Transcranial Electromagnetic Treatment

**FIGURE 3:**
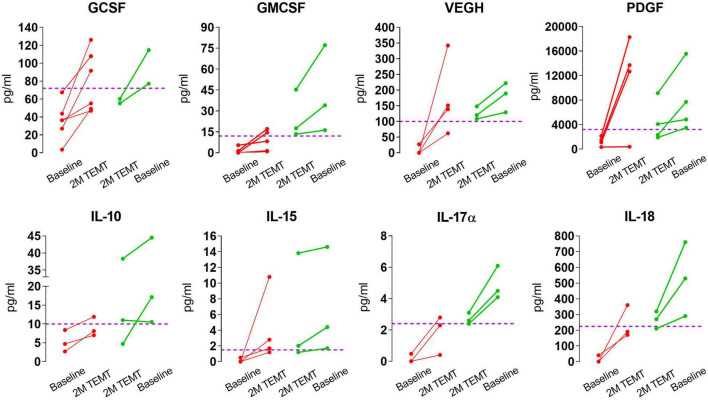
Effects of 2-months of daily TEMT on plasma levels of eight cytokines in AD subjects. As can be seen for all eight cytokines, if baseline plasma levels were low (below the horizontal “convergence” dashed line), TEMT resulted in increased blood levels after 2-months of treatment (red lines). Conversely, if baseline cytokine levels were high, TEMT resulted in decreased blood levels after 60 days of treatment (green lines). Both red and green data points after 2 months of TEMT largely gravitate to at or near the horizontal “convergence” dashed line. These results suggest a clear ability of TEMT to re-balance or attempt to rebalance plasma cytokine levels to normal.

For individual AD subjects, Figure 3 shows eight plasma cytokine responses to 2-months of daily TEMT compared to baseline. Subjects with lower baseline levels of a given cytokine in plasma showed increases in that cytokine as a result of treatment. By contrast, those subjects with higher baseline levels of a given cytokine show treatment-induced decreases.

**FIGURE 4:**
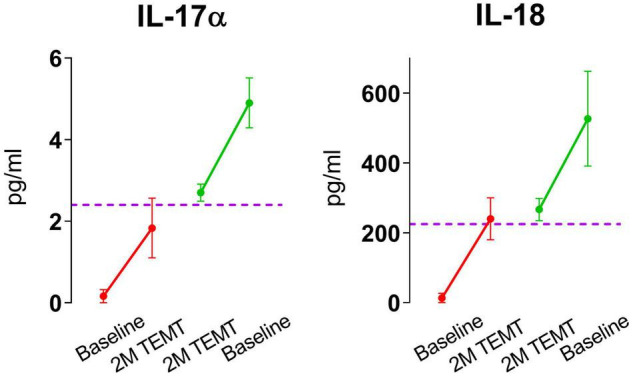
Means and SEMs for the individual subjects shown in Figure 3 for IL-17α and IL-18. As is evident for both cytokines, the response to TEMT is dependent on baseline levels, with TEMT inducing convergence (rebalancing) of both IL-17α and IL-18 toward aged normal levels (horizontal dashed line).

For all eight plasma cytokines shown in Figure 3, their levels following 2-months of TEMT had gravitated to or over-shoot a plasma level that was approximated for showing where individual responses appeared to be converging at 2-months (dashed horizontal line for each graph). Each cytokine’s approximate convergence level was at or near plasma levels previously reported in aged control (unimpaired) subjects (Table 4). This treatment-induced convergence of plasma cytokine values is vividly shown in Figure 4 for IL-17α and IL-18, wherein means and SEM’s are shown for the two opposite cytokine responses dependent on baseline cytokine levels. Figures 3, 4 repeatedly show an immunoregulatory or “rebalancing” action of TEMT to return, or attempt to return, plasma cytokine levels to the normal levels of aged, unimpaired individuals.

**TABLE 4:** Plasma cytokine levels in AD subjects converge to normal or near normal levels following TEMT.

Cytokine	Approximate convergence level after 2-months of TEMT (pg/ml)	Published normal aged level (pg/ml)
GCSF	72	73.6 Leung et al., 2013
GMCSF	12	20.3 Kim, J. Trans. Med. 2011; 9: 113
VEGF	100	100 Kim, J. Trans. Med. 2011; 9: 113
PDGF	3,200*	3320 Leung et al., 2013
IL-10	10	10.0 Leung et al., 2013
IL-15	1.5	2.0 Gangemi, Med. Inflam. 2005; 4: 245
IL-17α	2.4	2.0 Simundic, Kid. BP Res. 2017; 42: 99
IL-18	225	179 Oda, PLOS ONE 2013; 8: e81497

*Based only on 2-month levels in the higher baseline PDGF subjects (Group 2) because most subjects in the lower baseline group (Group 1) had appreciably over-shot normal levels.

**FIGURE 5:**
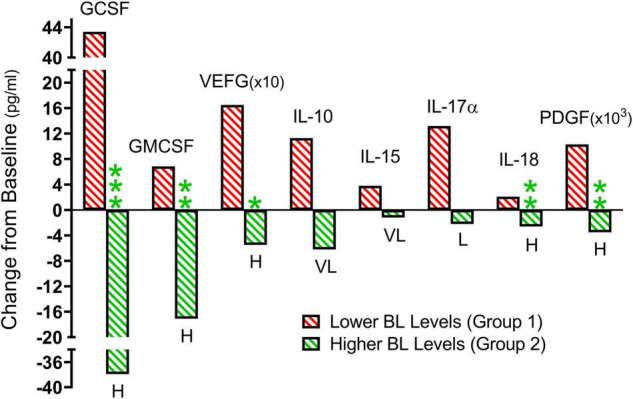
From the individual subject results presented in Figure 3, summarized means of TEMT-induced changes for Group 1 (red; lower baseline levels) and Group 2 (green; higher baseline levels). When comparing Group 1 to Group 2, the direction of plasma cytokine response to 2 months of TEMT is universally opposite for all eight cytokines. The difference is frequently significant (*p*-value) and there is a substantial Effect Size (clinically important effect) for all eight cytokines. For Group 1 vs. Group 2 significance: *p < 0.05, **p < 0.02, ***p < 0.001. For Group 1 vs. Group 2 Effect Size: L, large; VL, very large; H, huge.

Figure 5 shows the TEMT-induced changes in the eight cytokines in Figure 3, summarized with means for the treatment-induced change from baseline that occurred when AD patients were divided into two groups based on their plasma “baseline” cytokine levels being either lower (Group 1) or higher (Group 2). The direction of plasma cytokine response to TEMT is always opposite for these two groups and the difference is often significant with *t*-test (*p* < 0.05). Moreover, these opposite TEMT-induced changes between the low (Group 1) vs. high (Group 2) cytokine group were of clinical importance for all eight cytokines based of Effect Size, with Cohen’s *d* calculations revealing “very large” or “huge” clinically important differences for seven of the eight cytokines (Figure 5).

### Changes in Plasma Cytokine Levels Occur After Only a Single 1-h Transcranial Electromagnetic Treatment Administration

**FIGURE 6:**
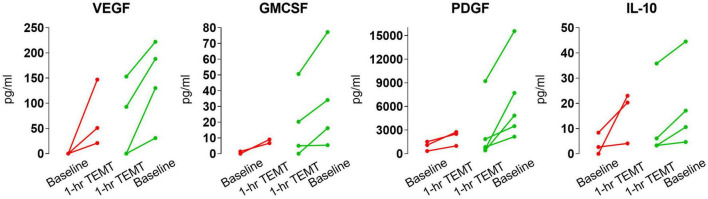
Effects of a single 1-h TEMT treatment on plasma levels of four cytokines in AD subjects. For all four cytokines, if baseline blood levels were low (red points and lines), 1-h of TEMT induced an increase in plasma levels in samples taken several hours thereafter on Day 1. Conversely, if baseline cytokine levels were high (green points and lines), 1-h of TEMT resulted in decreased blood levels shortly thereafter on Day 1.

The ability of TEMT to regulate plasma cytokine levels was even present after the initial 1-h of TEMT, with lower baseline cytokine levels at baseline resulting in higher levels after this single TEMT, and just the opposite occurring if baseline levels were higher (Figure 6). Indeed, this single TEMT was already inducing convergence of these four plasma cytokines to the convergence levels indicated in Table 4 for 2 months of TEMT. These immediate treatment-induced cytokine responses suggest a remarkable “acute” immunoregulatory action (rebalancing) of plasma cytokines by TEMT, in addition to TEMT’s long-term immunoregulatory actions after 2-months of treatment (Figures 3, 4).

### Baseline CSF Cytokine Levels Determine Direction and Extent of Response to 2 Months of Transcranial Electromagnetic Treatment

As was the case for plasma cytokine levels, there were no “overall” TEMT-induced changes in CSF levels of the seven cytokines that were above lower assay limits following 2-months of treatment (Table 5).

**TABLE 5:** Overall effects of 2-months TEMT administration on cytokines/growth factor levels (pg/ml) in CSF.

Cytokine	Baseline (BL)	Day 60	*p*-Value	*n*
GCSF	121.50 ± 31.73	116.11 ± 19.38	0.836	7
VEGF	27.2 ± 16.3	37.0 ± 30.8	0.831	4
IL-15	7.70 ± 1.35	8.30 ± 1.34	0.62	7
IL-17α	1.96 ± 0.12	1.95 ± 0.80	0.945	8
TGF-α	4.26 ± 0.40	4.18 ± 0.35	0.867	7
IL-8	57.57 ± 12.09	60.37 ± 15.41	0.652	8
NGF	0.47 ± 0.15	0.43 ± 0.19	0.666	7

**FIGURE 7:**
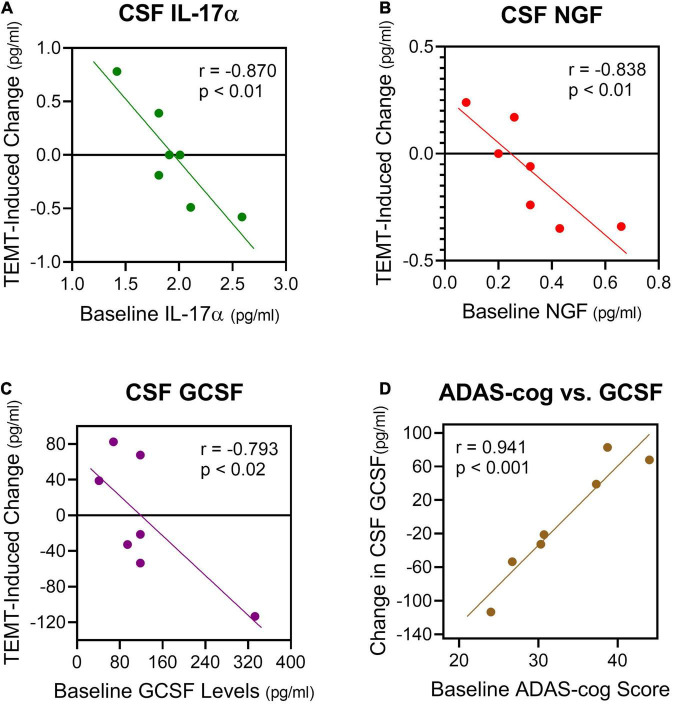
**(A–C)** For five cytokines in CSF, 2 months of TEMT increased their CSF levels if lower levels were present at baseline, and just the opposite for higher CSF levels at baseline. Three examples of the significant correlations resulting from this rebalancing of CSF cytokine levels by TEMT are presented. Thus, baseline CSF levels of at least five cytokines determine the direction and extent of cytokine response to TEMT. **(D)** Baseline ADAS-cog scores correlated strongly with the response of GCSF in CSF to TEMT.

Also very similar to the effects of TEMT on plasma cytokine levels (Figures 3, 4), the response of CSF cytokine levels to 2-months of TEMT for five of the seven CSF cytokines was dependent on whether baseline cytokine levels were lower or higher. Specifically, TEMT increased CSF cytokine levels if lower levels were present at baseline, while TEMT decreased CSF cytokine levels if higher levels were present at baseline. Significant correlations were present for all five of those CSF cytokines, as exemplified for three of them (IL-17α, NGF, and GCSF) in Figures 7A–C. Correlation coefficient values/significance for the other two cytokines (VEGF and TGFα) were (*r* = −0.806; *p* < 0.05) and (*r* = −0.716; *p* < 0.05), respectively. These significant correlations strongly suggest that baseline levels of at least five CSF cytokines determine the direction and extent of their response to TEMT. Even for the remaining two measurable cytokines in CSF (IL-8 and IL-15), the response to TEMT for all but one AD patient was dependent on whether baseline cytokine levels were lower or higher.

Although normal adult/aged CSF levels of cytokines in humans are apparently not well-published, the “convergence” CSF cytokine level (zero intercept) for IL-17α, NGF, and GCSF from Figures 7A–C would predict that normal CSF cytokine levels are 1.8, 0.25, and 140 pg/ml, respectively. For GCSF in CSF, baseline ADAS-cog score even correlated strongly with the direction and extent of the GCSF response to TEMT (Figure 7D). Higher (poorer) ADAS-cog scores at baseline were associated with increased GCSF levels in CSF, while just the opposite occurred for lower (better) ADAS-cog scores.

It should be noted that baseline CSF levels of four cytokines (GCSF, IL-8, IL-15, and TGFα) were much higher than their baseline plasma levels for all AD subjects separately and collectively. This signifies that cells within the brain were secreting at least these four cytokines and that TEMT affected the brain secretion of at least two of these four cytokines (GCSF and TGFα).

## Discussion

Age-related changes in the immune system are undisputable, with either hyper- or hypo-activation causing diseases. Whether AD involves over-activation (Fila, 2010; Rubio-Perez and Morillas-Ruiz, 2012; Sopova et al., 2014; Hu et al., 2019; Rea et al., 2019; Webers et al., 2020) or under-activation (Renzos et al., 2007; Laske et al., 2009) of the immune system, or a combination of both, is unresolved. In any case, the goal is for the immune system to maintain balance during aging, which is best indicated by cytokine levels. Therefore, a therapeutic intervention that “rebalances” the immune system’s cytokine levels to provide overall immune regulation in both brain and systemic circulation could contribute to an arresting or reversal of AD cognitive impairment.

Such a therapeutic appears to be TEMT, which we have previously shown to result in reversal of cognitive impairment in AD subjects after 2-months of daily treatment (Arendash et al., 2019). The present study strongly suggests that TEMT exerts an immunoregulatory function in AD subjects – one that seeks to achieve immunological “rebalancing” by returning high or low cytokine levels in AD subjects to the normal or near normal levels of unimpaired aged adults. Although cytokine levels in both plasma and CSF were similarly affected by TEMT, the relative importance of peripheral vs. brain cytokine modulation by TEMT remains unknown. Indeed, how the immune system in the brain and the immune system in the periphery are connected functionally is also unknown. The discovery in 2015 of lymphatic vessels in the human brain (Louveau et al., 2015) provided anatomic evidence that the immune system in the brain can indeed communicate with the peripheral/systemic immune system. These “meningeal lymphatic” vessels, lining the dura sinuses, are able to carry both fluid and immune cells from the cerebrospinal fluid to the deep cervical lymph nodes, then into the venous circulation.

The present results, in concert with other studies (Cao et al., 2012; Leung et al., 2013; Rea et al., 2019; Taipa et al., 2019), suggest that an imbalance of plasma/brain cytokines is contributory to development of AD and/or its progression. Thus, a “rebalancing” of the cytokine levels in both brain and peripherally should be most advantageous against AD. TEMT would appear to provide such a rebalancing of cytokine levels in both of these immune compartments. Immunoregulation can then be added to TEMT’s other two identified mechanisms of action in the AD brain: (1) disaggregation of Aβ and tau aggregates/oligomers (Arendash et al., 2010; Dragicevic et al., 2011), and (2) mitochondrial (energy) enhancement (Dragicevic et al., 2011; Arendash et al., 2019).

### Transcranial Electromagnetic Treatment Effects on Plasma Cytokines

A substantial body of both experimental and clinical research suggests an influential role of the systemic immune system in AD pathogenesis (Cao and Zheng, 2018), so changes in cytokine levels within plasma could play a role in development or prevention/treatment of the disease (Leung et al., 2013). In the present study, there were no “overall” effects of 2 months of daily TEMT on plasma cytokine levels for all eight mild/moderate AD subjects combined. However, our data analysis indicate that AD subjects could be divided into two groups from their baseline plasma cytokine levels and ADAS-cog scores. Group 1 consisted of AD subjects with sub-normal plasma cytokine levels at baseline, intimating they had been unable to mount an immune response to the disease and so were more cognitively impaired at baseline (poorer ADAS-cog scores). Group 2 consisted of AD subjects with higher-than-normal plasma cytokine levels at baseline, suggesting their immune system had still been fighting AD and so they were better in cognitive performance at baseline than Group 1 (though still cognitively impaired). For those AD subjects with low plasma cytokine levels at baseline, TEMT performed an important “rebalancing” function by re-activating/resurrecting plasma cytokine levels (both pro- and anti-inflammatory components). For those subjects with higher cytokine levels at baseline, the decreases induced by TEMT also resulted in normal or near normal cytokine levels (i.e., an immune rebalancing). Both groups showed cognitive improvement after 2 months of daily TEMT. It is important to underscore that these rebalancing effects of TEMT on the peripheral immune system occurred in an array of at least 9 of the 12 cytokines evaluated, with each affected cytokine being either pro- or anti-inflammatory, or both. Thus, not just a single target cytokine was affected, such as for the drugs that are being developed to selectively block TNF-alpha (Decourt et al., 2017).

Since TEMT can apparently re-activate/resurrect a hypoactive immune system in AD subjects – subjects who showed concurrent cognitive benefits from TEMT (Arendash et al., 2019) – it is reasonable to anticipate that MCI patients having low plasma cytokine levels would also benefit from TEMT, with a re-activation of their peripheral immune system. More specifically, we propose that a hypo-active peripheral immune system (i.e., sub-normal plasma cytokine levels) in MCI patients is insufficient for them to avoid or fight off progression to AD. Indeed, we have direct evidence for this view in that MCI subjects who converted to AD over a 2–4 years observation period had *sub-normal* plasma cytokine levels at study initiation. By contrast, MCI subjects who remained stable in MCI (i.e., no conversion to AD) over that period had *normal* levels of plasma cytokines (Cao et al., 2012). Therefore, it is likely that TEMT to MCI patients would re-balance their low plasma cytokine levels to normal, thus preventing or delaying MCI conversion to AD. This extraordinary capacity of TEMT to conceivably prevent or delay MCI conversion to AD will be the subject of our future clinical trials.

Of the plasma cytokines/growth factors regulated by TEMT, it is easy to list the potential benefits that five of them (GCSF, GMCSF, VEGF, IL-10, and PDGF) can provide. However, the remaining cytokines (IL-15, IL-17α, and IL-18, and IFN-γ) are largely pro-inflammatory. So for the subjects that started out with low baseline levels of these pro-inflammatory cytokines, treatment induced increases in their plasma levels in addition to the five other cytokines. We believe this should not be looked at as deleterious since these cytokines can perform useful functions against AD (i.e., stimulate microglial cells to remove Aβ) and contribute to rebalancing of the peripheral immune system by TEMT (Cao et al., 2012; Leung et al., 2013; Rea et al., 2019; Taipa et al., 2019). Therefore, the 5–6 subjects who started with low baseline levels of all 8 cytokines got a “balanced” re-activation (strengthening) of both pro- and anti-inflammatory components in plasma, which may have contributed to the reversal of cognitive impairment seen in these same AD subjects (Arendash et al., 2019).

### Transcranial Electromagnetic Treatment Mechanism of Plasma Cytokine Action

**FIGURE 8:**
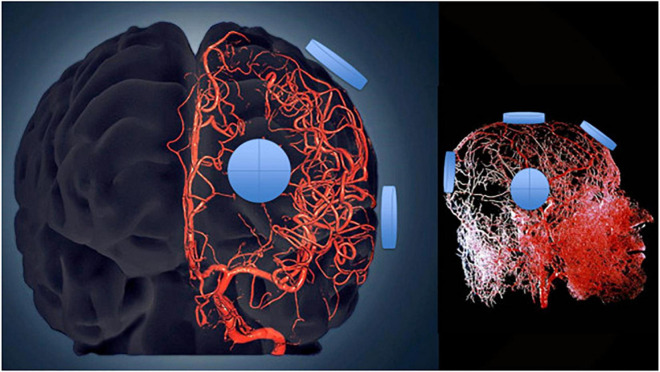
Three-dimensional model of the cerebral arterial tree showing the location of four emitters on the surface of the head’s right side for the MemorEM device. The emitters (blue disks) are in close proximity to affect all blood components within the cerebrovascular tree including arteries/arterioles and most importantly capillaries, where the velocity of blood cells and plasma is at its slowest for maximal EMF exposure.

A single initial 1-h treatment was enough to already affect plasma cytokine levels and in the direction to be seen for each cytokine after 2 months of treatment – namely, that lower baseline levels and poorer baseline ADAS-cog performance resulted in higher Day 1 levels, with just the opposite occurring if baseline cytokine levels were higher and baseline ADAS-cog performance better. These immediate treatment-induced cytokine responses strongly suggest an “acute” immunoregulatory action of TEMT in addition to its long-term regulatory actions after 2-months of treatment. It is possible that both acute and long-term responses to TEMT involve the same mechanism(s) of action. Mechanistically, TEMT is most likely providing its rebalancing or immunoregulatory effects on plasma cytokines by affecting blood cells traveling through the brain’s dense vascular system, particularly in the brain’s capillaries where blood flow is the slowest and thus most exposed to EMF effects (Figure 8). In this regard, WBCs secrete a wide variety of cytokines that are exposed to electromagnetic waves, particularly as they go through capillaries in the brain. However, whole body 900 MHz at 1–2 W/kg SAR in mice daily for up to 4 weeks had no effect on multiple lymphocyte measures, such as on T and B cells, or serum levels of IgM and IgG (Gatta et al., 2003; Nasta et al., 2006). Alternatively, the brain’s microglia, astrocytes, and choroid plexus secrete cytokines (Stopa et al., 2001; Brillaud et al., 2007; Kudo et al., 2007; Ammari et al., 2008; Yang et al., 2010) and thus may have impacted plasma cytokine levels for those cytokines in higher concentration within CSF compared to plasma.

### Transcranial Electromagnetic Treatment Effects on CNS/CSF Cytokines

No “overall” effects of 2 months of TEMT were seen on CSF levels of the seven cytokines that were measurable. However, TEMT did induce changes in five of these seven CSF cytokines (IL-17α, NGF, GCSF, VEGF, and TGFα) that were significantly correlated with their baseline cytokine levels – just as was the case for plasma cytokines. For each of these cytokines, lower CSF levels at baseline resulted in TEMT-induced “increases” in their CSF levels, while just the opposite occurred if higher baseline levels were present in CSF. Even the remaining two CSF cytokines showed the same TEMT-induced changes, except that one subject for each cytokine did not fit the overall pattern. Thus, TEMT sought to re-balance levels of these CSF cytokines, with the rather predictable direction of this rebalancing being dependent on their baseline levels. For GCSF in CSF, even ADAS-cog scores were correlated with direction and extent of TEMT-induced responses.

Surprisingly, normal CSF levels for aged individuals have apparently not been well-reported in the scientific literature. Therefore, a determination of “convergence” to normal CSF levels by TEMT was not possible for the present study.

Importantly, CSF levels of GCSF (and TGFα) were much higher than their plasma levels in all eight subjects. Therefore, it is clear that cells in the AD brain are secreting GCSF and that their secretion of GCSF is affected by TEMT administration (see next section). We have previously reported that AD transgenic mice given 3 weeks of GCSF or GMCSF injections showed CNS enhancements in hippocampal neurogenesis, microglial activation/Aβ degradation, and synaptogenesis (Sanchez-Ramos et al., 2009; Boyd et al., 2010). In the present study, these three beneficial effects would be anticipated to be enhanced by TEMT in those AD subjects who had low baseline GCSF levels in CSF, all of whom showed TEMT-induced increases in CSF levels of GCSF. Parenthetically, for those AD subjects with higher baseline GCSF levels in CSF, their TEMT-induced decreases still resulted in appreciable GCSF levels in CSF.

### Transcranial Electromagnetic Treatment Mechanism of CNS/CSF Cytokine Action

Since these are the first reported effects of TEMT on CSF cytokine levels in humans, the exact mechanism(s) of TEMT’s effects on brain cytokine levels in AD subjects is currently unknown. Indeed, more studies are needed to adequately characterize differences in CSF cytokine levels of AD subjects compared to healthy controls (Hu et al., 2019). Microglia, as well as astrocytes, both secrete cytokines (Lu et al., 2014), so TEMT may be affecting cytokine secretion from one or several of these CNS cell types. Stimulated/activated microglia and astrocytes undergo a pro-inflammatory response by releasing inflammatory cytokines (Hanisch and Kettenmann, 2007; Tribouillard-Tanvier et al., 2012; Medeiros and LaFerla, 2013). In this regard, previous studies have reported electromagnetic wave effects on both microglia and astrocytes. Specifically, brain microglial activation was histologically observed in rats following their exposure to 915 MHz (Kudo et al., 2007). Similarly, microglia in cell culture given a single 20 min EMF exposure at 2450 MHz were activated, resulting in a pro-inflammatory secretion of TNFα (Yang et al., 2010). Astrocytes in cell culture have been reported to be activated by exposure to 900 MHz electromagnetic waves (Brillaud et al., 2007; Ammari et al., 2008). These generally acute EMF exposure studies in cell cultures or rodents appear to indicate the ability of 900 MHz EMF to activate both brain microglia and astrocytes. However, Thorlin et al. (2006) reported no effect of 900 MHz electromagnetic waves at similar power levels on release of several cytokines from astrocyte cell cultures or on microglial cell activation in culture. An alternative mechanism of CNS/CSF cytokine regulation by TEMT may be it affecting choroid plexus epithelial cells or macrophages resident within the choroid plexus (Stopa et al., 2001; Cui et al., 2021). The choroid plexus epithelial cells in particular secrete cytokines into the CSF that they produce (Stopa et al., 2001). The effects (if any) of electromagnetic waves on secretion of cytokines into CSF from such epithelia cells is unknown. Therefore, which of these three brain cell types (microglial, astrocytes, and/or choroid plexus epithelial cells) are responding to TEMT by seeking to “rebalance” multiple cytokines in the CSF and/or brain is an important open question for future investigations. In that regard, we are currently using cell cultures to determine the effects of TEMT on various CNS and peripheral cell types.

### Study Limitations

There are several limitations to this study that should be indicated. First, the study only involved AD subjects given TEMT. The cytokine responsivity of normal, unimpaired subjects to TEMT would be important to determine if similar or difference responses to treatment are observed. We would predict that, if a given cytokine’s level in such subjects is within normal range, that TEMT would not affect that cytokine’s level. We saw evidence of this in the present study, wherein TEMT to subjects whose baseline cytokine levels in plasma were near normal showed a minimal or no treatment response.

Second, a larger group of mild/moderate AD subjects would have been highly desirable (including placebo controls), despite the numerous significant and consistent TEMT-induced effects demonstrated for the eight AD subjects in this study. It is important to underscore that multiple cytokines were investigated in both plasma and CSF, with the same directional result of TEMT for 12 of them depending on their initial levels. For example, 55 individual subject opportunities for any given cytokine in plasma to increase or decrease after TEMT resulted in all 55 being predicted correctly from their initial baseline levels – the chances of this happening by chance are 1 in 3.6 × 10^16^. As well, most statistically significant effects reported in this study occurred at “*p*” levels of 0.02 or higher level of significance. Thus, a reproducibility of TEMT effects across multiple cytokines in both plasma and CSF was observed, providing enhanced confidence that the presented results are indeed reliable and the conclusions based on those results sound.

The relatively small number of AD patients in the present study (*n* = 8) was the result of both our high bar for confirmation of AD and the difficulty of recruiting AD subjects at the USF Byrd Alzheimer’s Institute at the time, which had multiple other clinical trials requiring recruitment at the same time. The sheer number and labor-intensity of end-points in this initial Pilot study also contributed to the relatively small number of subjects involved. Also regarding the number of subjects in this study, it should be noted that initial (Pilot) studies involving neuromodulatory approaches against AD (such as transcranial magnetic stimulation and deep brain stimulation) have typically involved a small number of subjects (less than 10). This study’s comprehensive design and the presence of both cognitive and non-cognitive benefits/effects strengthen the premise that TEMT had a real and meaningful impact in the study’s AD subjects. Given the enthusiasm of both AD patients and their caregivers following this study’s 2-month treatment period, the study has been extended to a 31-month (21/2 year) treatment period. Also, as a direct follow-up to this initial 2-month clinical trial, a Phase IIb (Pre-Pivotal) clinical trial is underway that involve 34+ AD subjects in three groups (high TEMT, lower TEMT, and placebo), with TEMT given daily for 5 months.

Third, we only evaluated plasma/CSF cytokines and not blood cells (e.g., T cell function/numbers, complete blood cell counts with differential). However, mice given similar EMF treatment daily for up to 4 weeks did not show any changes in multiple lymphocyte measures or serum IgG levels (Gatta et al., 2003; Nasta et al., 2006). Fourth, the immune system may have adjusted or compensated to TEMT over the daily 2 month (long-term) treatment in this study in a hormetic fashion. However, we saw no evidence of this since both acute (one treatment) and long-term cytokine responsivity were the same.

## Conclusion

Results from the present study strongly suggest that wide-spread immunoregulation or “rebalancing” of both vascular/peripheral and brain/CSF cytokine levels can be accomplished with TEMT, both acutely and long-term. The extraordinary ability of TEMT to modulate cytokine levels in both central and peripheral immune compartments could provide for a coordinated immune rebalancing within and between both compartments. Such immunoregulation can be added to the two already-identified mechanisms of TEMT action against AD (toxic oligomer disaggregation, mitochondrial enhancement). These three actions of TEMT against AD collectively provide a safe, non-drug cocktail of mechanisms that may be providing an unparalleled, multi-targeted attack against AD – an attack that may very well be responsible for the reversal in cognitive impairment observed in the same AD patients. Our results have broad implications for therapeutic intervention against a variety of both brain and body diseases/disorders wherein an immune imbalance exists.

## Data Availability Statement

The raw data supporting the conclusions of this article will be made available by the authors, without undue reservation.

## Ethics Statement

The studies involving human participants were reviewed and approved by the Western Institutional Review Board (now WCG-IRB). The patients/participants provided their written informed consent to participate in this study.

## Author Contributions

GA, CC, and HA designed the study. RB designed and fabricated the MemorEM devices. GA searched the literature and wrote the manuscript draft. HA, XL, NS, YW, and XZ collected the data. GA and XZ analyzed the data. GA and CC interpreted the data. All authors revised the manuscript and approved it for submission.

## Conflict of Interest

The University of South Florida has a financial interest in NeuroEM Therapeutics, a company that provided all of the financial support for this clinical trial. The interest has been reviewed and managed by the University in accordance with its Institutional Conflict of Interest policy. GA has a financial interest in NeuroEM Therapeutics as a common shareholder and has received a salary at times as CEO of the company. RB, as an employee of Left Coast Engineering, has received consulting and manufacturing fees from NeuroEM for a variety of services related to the MemorEM device and patent applications. He also owns common shares in NeuroEM. CC, XL, NS, and XZ were employed by the company MegaNano Biotech, Inc. The remaining authors declare that the research was conducted in the absence of any commercial or financial relationships that could be construed as a potential conflict of interest.

## Publisher’s Note

All claims expressed in this article are solely those of the authors and do not necessarily represent those of their affiliated organizations, or those of the publisher, the editors and the reviewers. Any product that may be evaluated in this article, or claim that may be made by its manufacturer, is not guaranteed or endorsed by the publisher.

## Footnotes

1
https://clinicaltrials.gov/ct2/show/NCT02958930
